# Comprehensive overview of antibody drug-related clinical studies in gynecology: insights from ClinicalTrials.gov

**DOI:** 10.3389/fmed.2025.1521587

**Published:** 2025-05-09

**Authors:** Xiaoling Zhou, Li Xiao, Fan Lai, Wei Chen, Congrong Zhou, Yi Deng, Tao Wang, Shasha Xing, Haoyang Diao, Mi Tang, Wenmei Guo, Erdan Luo

**Affiliations:** ^1^Department of Pharmacy, Chengdu Women's and Children's Central Hospital, School of Medicine, University of Electronic Science and Technology of China, Chengdu, China; ^2^Department of Medical Affairs, Chengdu Women's and Children's Central Hospital, School of Medicine, University of Electronic Science and Technology of China, Chengdu, China; ^3^Department of Obstetrics, Chengdu Women's and Children's Central Hospital, School of Medicine, University of Electronic Science and Technology of China, Chengdu, China; ^4^Department of Traditional Chinese Medicine, Chengdu Women's and Children's Central Hospital, School of Medicine, University of Electronic Science and Technology of China, Chengdu, China; ^5^Department of Good Clinical Practice, Chengdu Women's and Children's Central Hospital, School of Medicine, University of Electronic Science and Technology of China, Chengdu, China

**Keywords:** Clinicaltrials.gov, antibodies, gynecology, antibody–drug conjugates, bispecific antibodies, Fc-fusion proteins, administration route

## Abstract

Antibodies have been widely used globally over the past decade and play an increasingly important role in modern medicine. Notably, significant advancements have been achieved in the realm of gynecology, particularly in gynecological cancers. This study endeavors to present a thorough overview of antibody-related drug clinical studies in gynecology registered on ClinicalTrials.gov, focusing on the basic characteristics of trials, geographical distribution, administration routes, indications, and targets. The analysis indicates a rising prevalence of antibody–drug conjugates (ADCs), bispecific antibodies, and Fc-fusion proteins. This study will help develop new ideas for future research on antibodies in gynecology.

## 1 Introduction

Antibody therapeutics have emerged as one of the most prominent sectors in the biopharmaceutical industry. Since the approval of the first monoclonal antibody in 1986, the field has experienced rapid and substantial expansion (1). The development of antibodies has evolved from initial mouse-derived forms to chimeric, humanized, and fully human antibodies (2). They have already made a significant impact on the overall sales of biopharmaceutical products in recent years (3).

Antibody-related drugs exhibit high specificity for their target sites and demonstrate superior efficacy compared to conventional drugs, with fewer side effects (4). These agents have been widely applied in gynecology, especially for the treatment of gynecological cancers (5–7). For instance, bevacizumab combined with paclitaxel and carboplatin has been considered first-line therapy for patients with primary peritoneal cancer, fallopian tube cancer, or epithelial ovarian cancer at stage III or IV (8). Furthermore, the results of the KEYNOTE-826 study (9) reveal that pembrolizumab in conjunction with chemotherapy, with or without bevacizumab, is recommended as a category 1 therapy for patients with metastatic, recurrent, or persistent cervical cancer (10). Additionally, an increasing number of antibodies are currently under clinical trial evaluation (11–13). Despite these advancements, there is a lack of research providing a comprehensive overview of clinical trials involving antibody-related drugs in gynecology. To address this gap, registered clinical trials from ClinicalTrials.gov are reviewed in this study to provide new insights for future research into antibodies in gynecology.

## 2 Materials and methods

### 2.1 Data sources

The keywords “antibody” and “marketed antibody-related drugs in gynecology” were used to thoroughly search data on ClinicalTrials.gov. This was identified from the databases of the European Medicines Agency (EMA) and the United States Food and Drug Administration (FDA). Subsequently, entries containing “antibody” and specific terms—“Denosumab,” “Bevacizumab,” “Pembrolizumab,” “Romosozumab,” “Dostarlimab,” “Tisotumab vedotin,” or “Mirvetuximab soravtansine”—were retrieved in the field of “other terms.” The search was restricted to studies in “Early Phase 1, Phase 1, Phase 2, Phase 3, and Phase 4” with a study initiation date before 27 May 2024. Following the identification of all the keywords, the data were consolidated and duplicate entries were removed.

**Figure 1:**
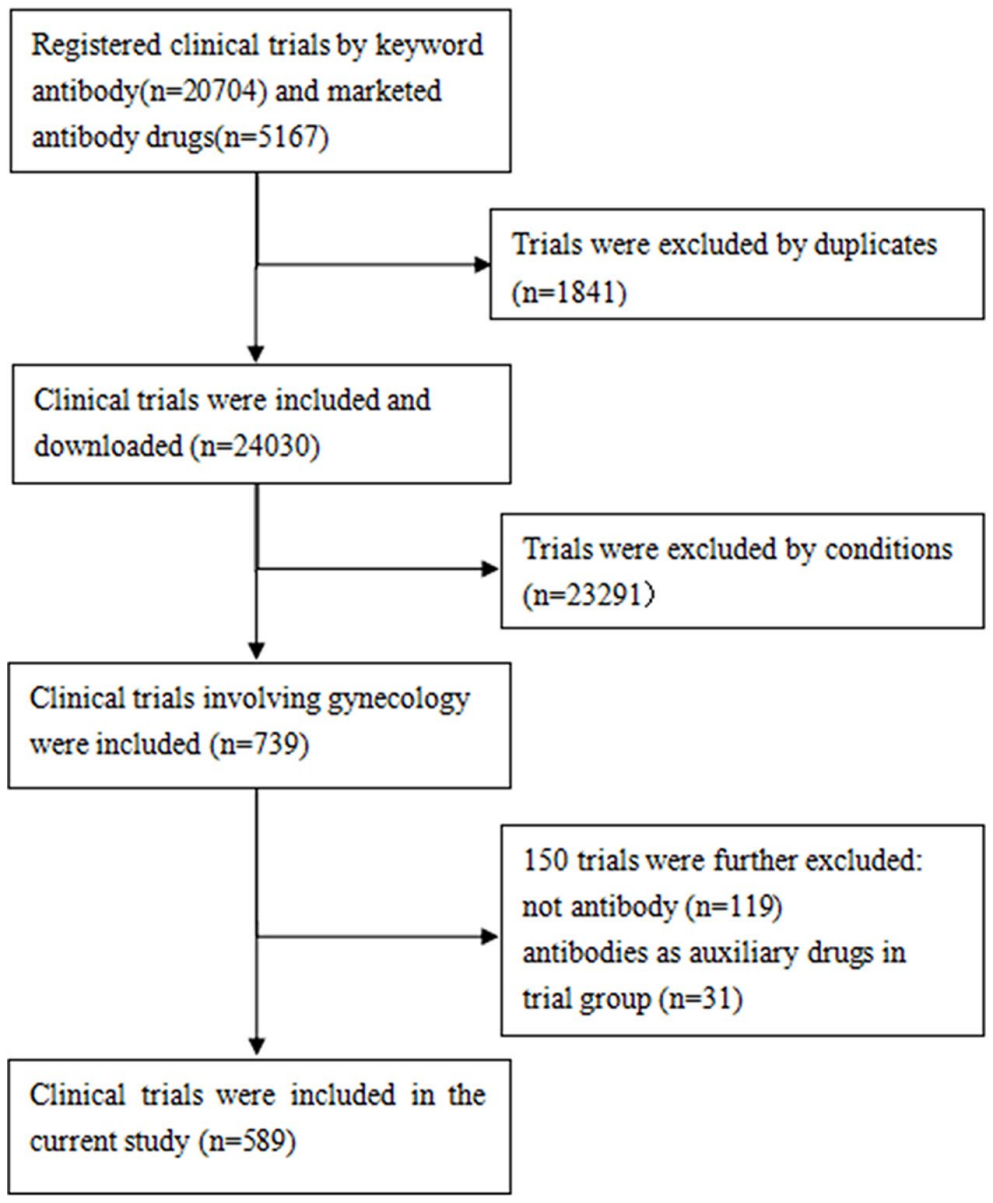
Process of determining the included trials.

The protocols established in previous investigations of ClinicalTrials.gov were followed (14–16). A comprehensive manual review of all Medical Subject Headings (MeSH) and disease terms cataloged in the National Library of Medicine was conducted to identify those relevant to gynecology. Subsequently, the identified terms were used to extract potential gynecological clinical trials. The study conditions were manually reviewed by a team of trained researchers to select gynecology-related trials. The exclusion criteria were as follows: (1) studies in which antibodies were used as control drugs, and (2) clinical trials in which antibodies were utilized as adjunctive therapies, with a major focus on assessing other drugs rather than the antibodies themselves. Ultimately, a total of 589 clinical trials were included. The data retrieval process is illustrated in Figure 1.

A variety of data were collected for analysis, including study title, condition, status, start date, trial phase, intervention, funder type, location, enrollment, administration route, and drug target. This study was not subjected to review by the institutional review board, since it used publicly available data without involving personal information.

### 2.2 Statistical analysis

Descriptive analysis was conducted using numerical and percentage formats for categorical variables, while data processing and analysis were performed via Microsoft Excel. Furthermore, network analysis was performed using Gephi to determine the locations and partnerships of the principal research institutions involved in the trials.

## 3 Results

### 3.1 Basic characteristics

**Figure 2:**
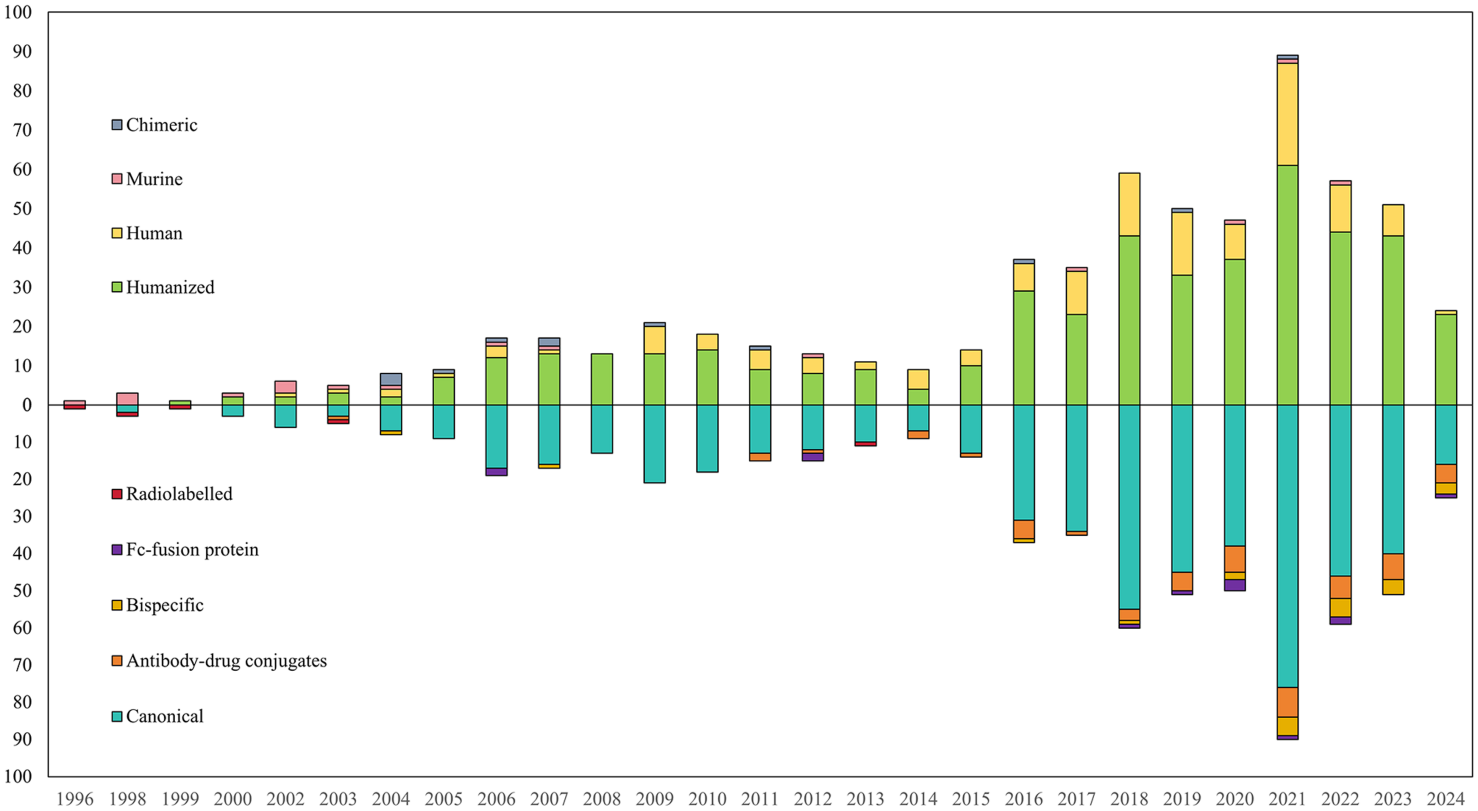
Annual number of different formats of antibody-related drugs in gynecology until May 27, 2024.

**Figure 3:**
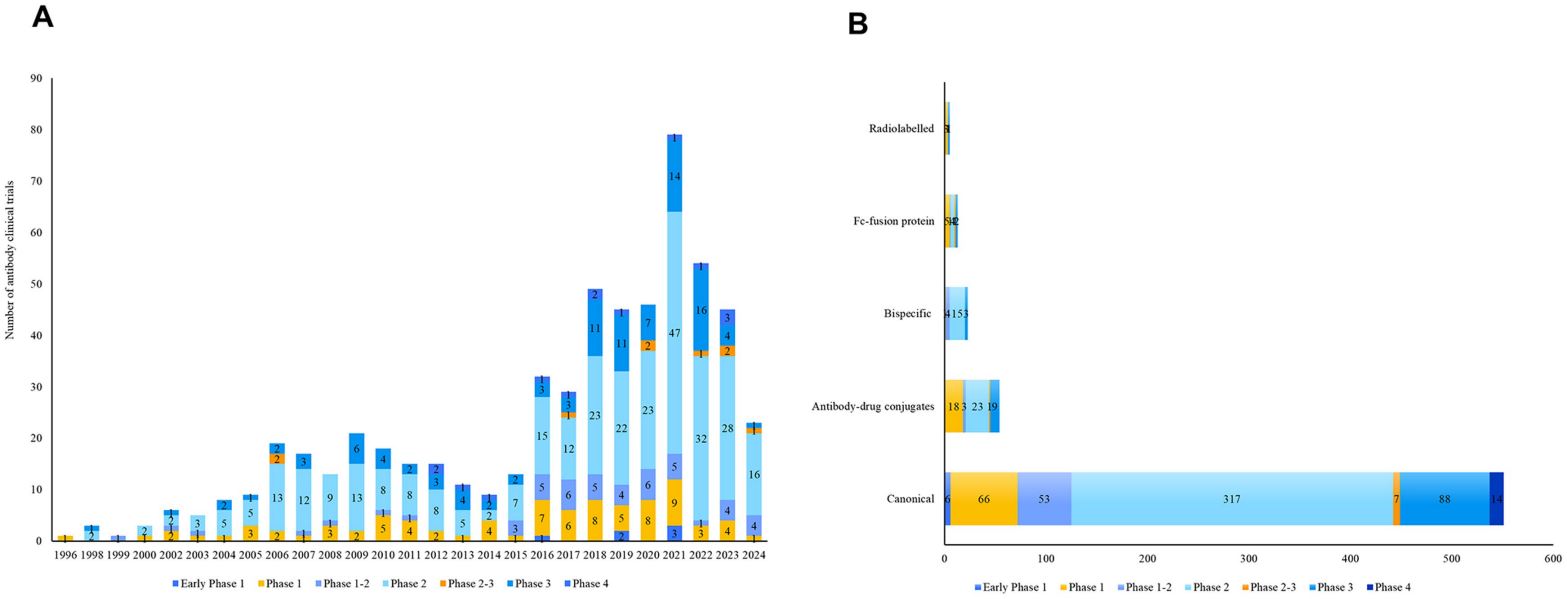
Overview of antibody trials. **(A)** The number of trials in each phase per year from 1996 to 2024; **(B)** the number of trials in each phase in various antibody types.

A total of 589 clinical trials involving antibody-related drugs in gynecology were analyzed, including 2 basket trials and 2 umbrella trials, which encompassed 646 antibody drugs. The fundamental characteristics of these trials are presented in Table 1. The antibody-related drugs investigated comprised 17 (2.69%) murine, 12 (1.90%) chimeric, 458 (72.35%) humanized, and 146 (23.06%) human antibodies. It is important to note that Fc-fusion proteins were not included. Approximately half of the studies (291, 49.41%) had planned or actual enrollment of 50 participants or fewer. The majority of antibody drug trials were funded by sources categorized as “other,” comprising 308 studies (52.29%). Meanwhile, 236 studies (40.07%) received funding from industry sources, and 45 studies (7.64%) were funded by the National Institutes of Health (NIH). Furthermore, all antibody-related drugs were classified into five categories: canonical antibody, ADCs, Fc-fusion protein, and bispecific and radiolabeled antibody. Figure 2 depicts the annual distribution of these different antibody-related drug formats, while Figure 3 gives an overview of antibody clinical research.

**Table 1:** Basic characteristics of trials.

Characteristic	No. (%)
Types of antibodies	
Murine	17(2.69)
Chimeric	12(1.90)
Humanized	458(72.35)
Human	146(23.06)
No. of site	
1	239(40.58)
≥2	327(55.52)
Not reported	23(3.90)
Enrollment of patients	
≤50	291(49.41)
51–100	110(18.68)
101–500	139(23.60)
501–1,000	37(6.28)
1,001–10,000	12(2.04)
Funding source	
NIH	45(7.64)
Industry	236(40.07)
Other	308(52.29)
Recruitment status	
Completed	213(36.16)
Active, not recruiting	87(14.77)
Not yet recruiting	31(5.26)
Recruiting	132(22.41)
Suspended	2(0.34)
Terminated	50(8.49)
Unknown status	55(9.34)
Withdrawn	19(3.23)
Phase	
Early Phase 1	6(1.02)
Phase 1	85(14.43)
Phases 1–2	50(8.49)
Phase 2	322(54.67)
Phases 2–3	9(1.53)
Phase 3	103(17.49)
Phase 4	14(2.38)
Interventional mode	
Parallel	243(41.26)
Single group	303(51.44)
Sequential	31(5.26)
Crossover	1(0.17)
Factorial	1(0.17)
Not reported	10(1.70)	Characteristic	No. (%)
Allocation	
Randomized	230(39.05)
Non-randomized	65(11.04)
Not reported	294(49.92)
Masking	
Open	491(83.36)
Single	8(1.36)
Double	32(5.43)
Triple	17(2.89)
Quadruple	37(6.28)
Not reported	4(0.68)

Percentages may not sum to 100% due to rounding. Fc-fusion proteins are not included in different types of antibodies.

### 3.2 Geographical distribution

**Figure 4:**
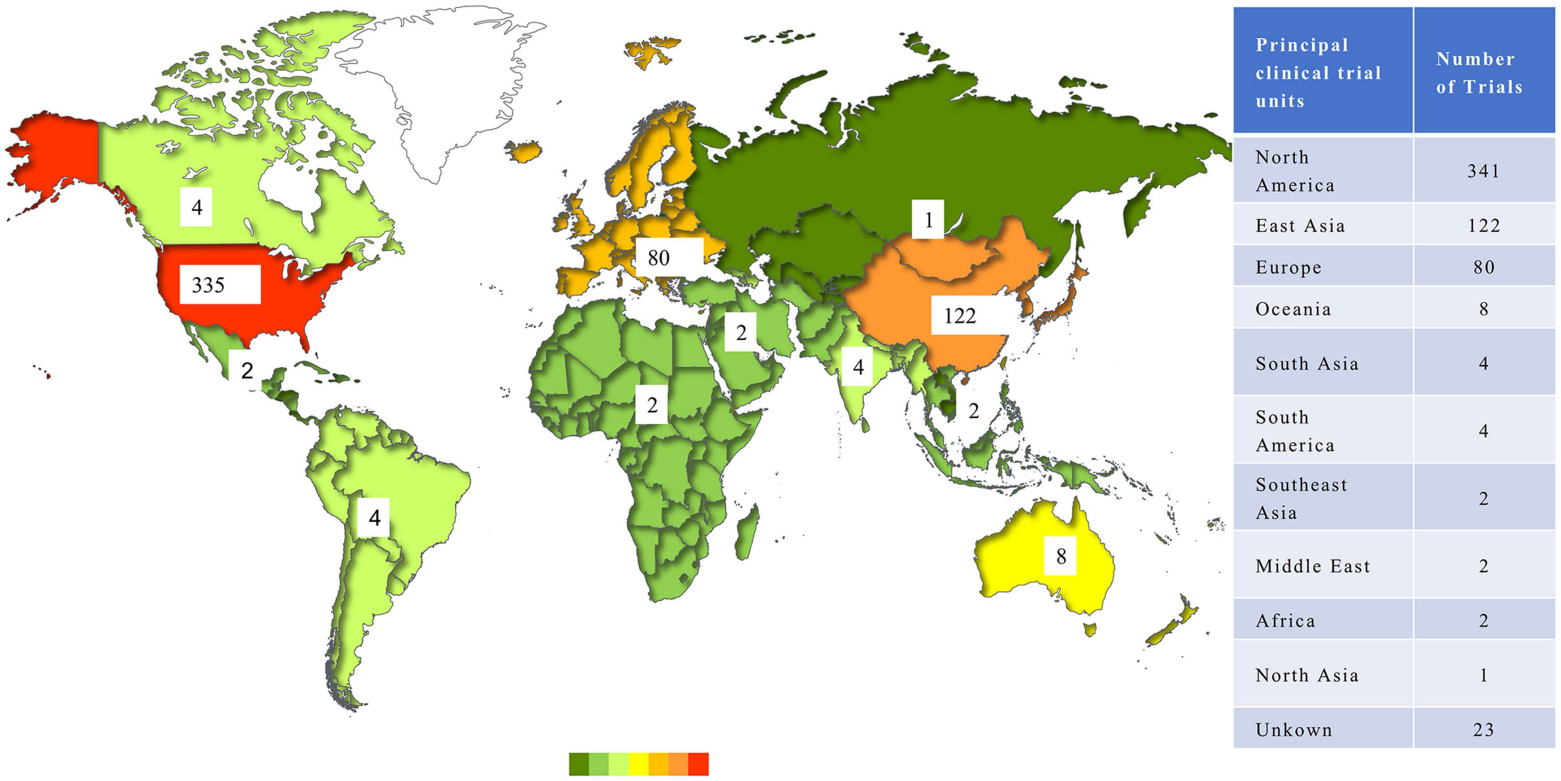
Distribution of clinical trials until May 27, 2024.

There were 566 trials conducted by 32 countries after the exclusion of trials lacking geographical information. In terms of regional distribution, North America enrolled the largest number of antibody trials (341, 57.89%), followed by East Asia (122, 20.71%) and Europe (80, 13.58%), while other regions had limited trials on this issue. As for countries, the United States possessed an obvious advantage in the development of antibody-related drugs, conducting 335 trials, which accounted for more than half of the world. China represented the most in Asia, with 104 trials. Figure 4 shows the distribution of clinical trials.

**Figure 5:**
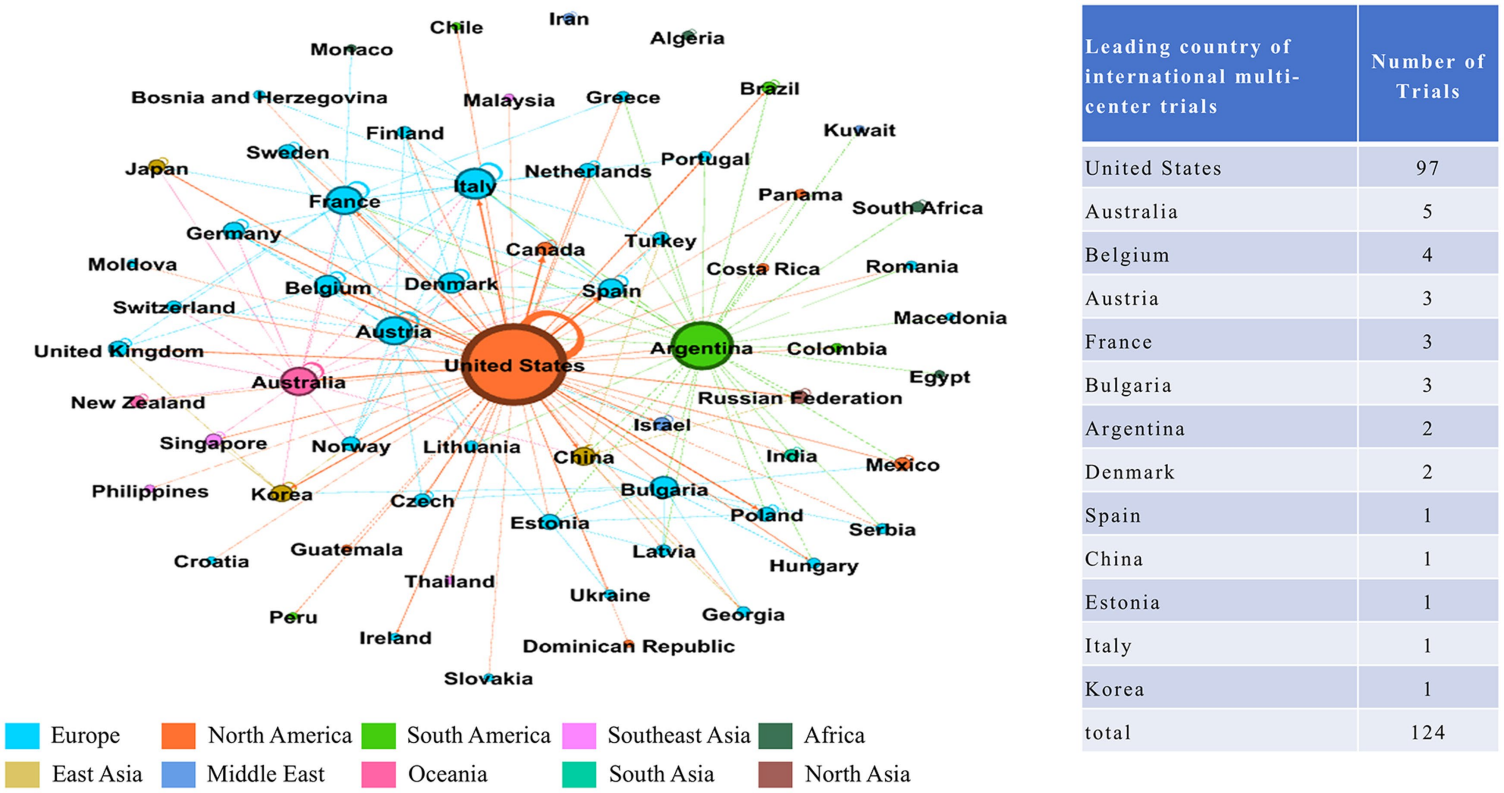
International cooperation network of trials. Each node represented a country, and the edge showed the cooperate connection between countries. The size of node represented the frequency of cooperation. Regions were represented by different colors.

In addition to the aforementioned leading countries, there were 124 trials conducted across multiple countries, which illustrated a network of international cooperation among trial sites. A total of 63 countries participated in these international multi-center trials, of which the United States served as the leading site in 97 instances and collaborated with up to 24 different countries. The primary collaborators with the United States included Australia (17 trials), Canada (16 trials), and Belgium (15 trials). Figure 5 illustrates the interconnection between the participating countries.

### 3.3 Administration routes

**Figure 6:**
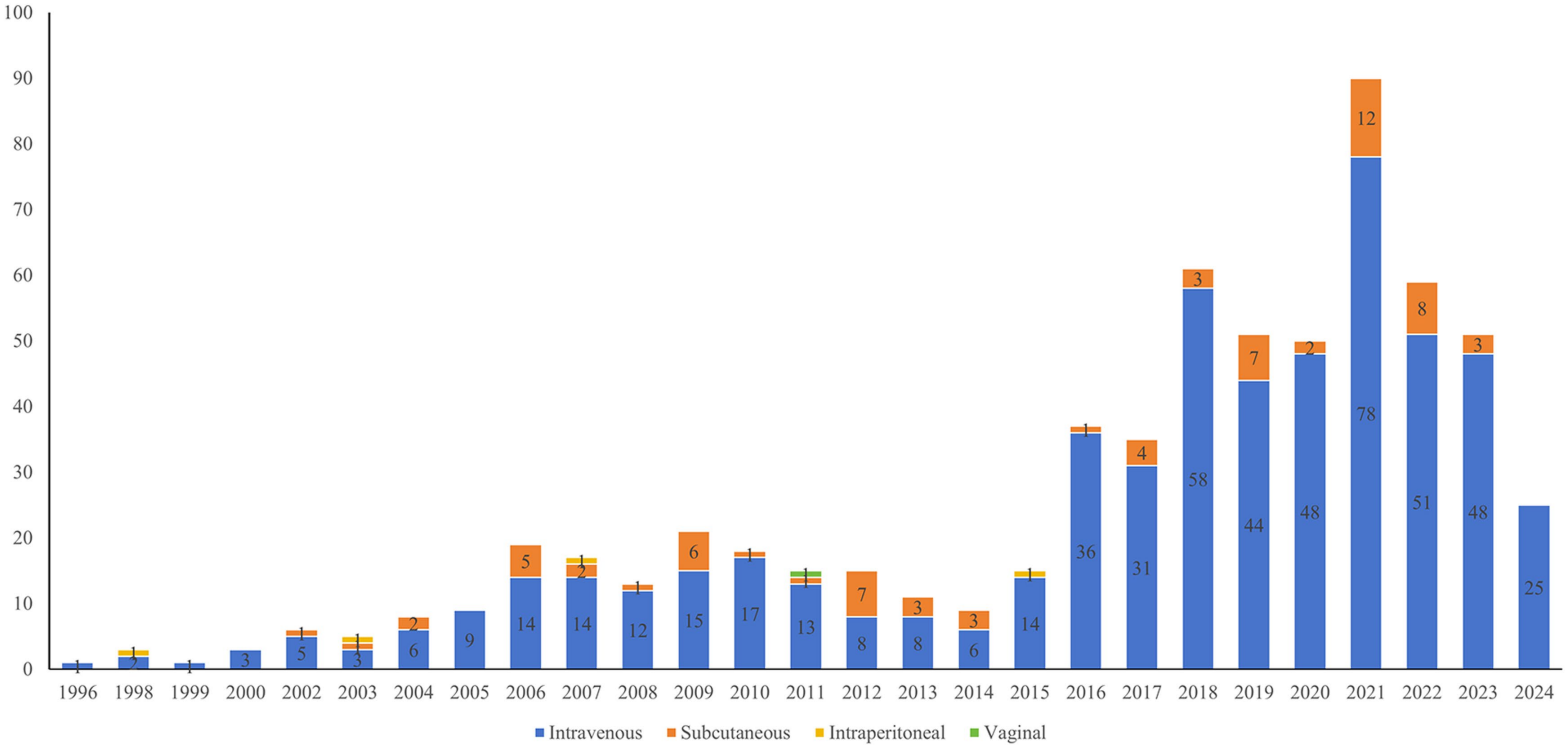
Annual number of antibody drug clinical trials in gynecology by drug administration until May 27, 2024.

Intravenous (IV) injection constituted the predominant method of antibody-related drug administration, which represented 568 cases (87.93%). Additionally, Subcutaneous (SC) injection was utilized in 72 cases (11.15%), intraperitoneal injection in 3 cases (0.46%), and vaginal administration in 1 case (0.15%). It is worth noting that two clinical trials used dual administration routes: combined IV and SC administration, and combined IV and intraperitoneal administration. Figure 6 illustrates the annual number of registered clinical trials categorized by drug administration.

### 3.4 Indications

**Figure 7:**
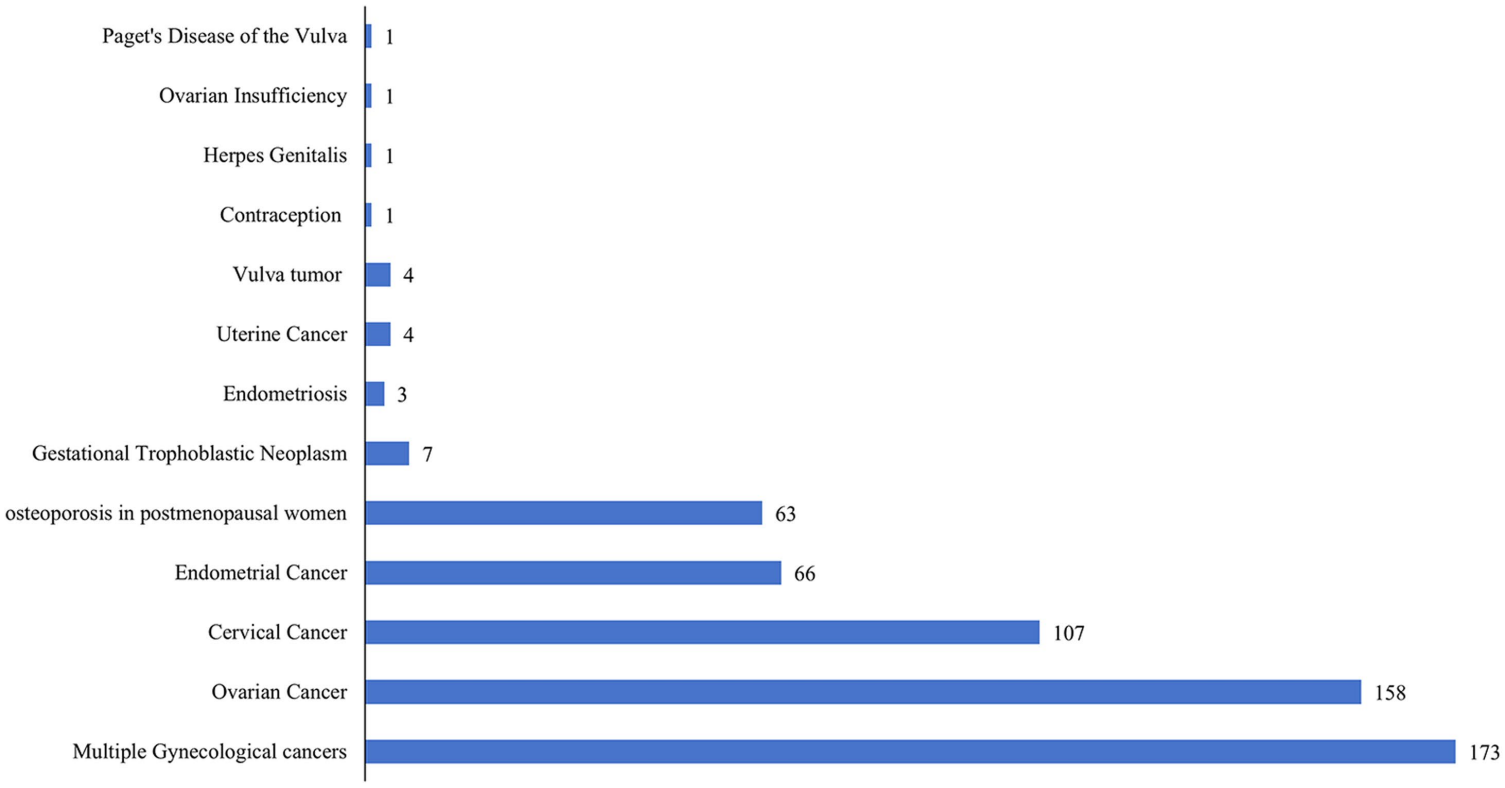
Number of new antibody drug clinical trials in gynecology by indications until May 27, 2024.

Antibodies utilized for treating gynecological cancers were predominantly represented in clinical studies. These included ovarian cancer (158, 26.83%), cervical cancer (107, 18.17%), endometrial cancer (66, 11.21%), gestational trophoblastic neoplasm (7, 1.19%), uterine cancer (4, 0.68%), vulvar tumor (4, 0.68%), Paget’s disease of the vulva (1, 0.17%), and multiple cancers (173, 29.37%). Furthermore, antibodies were used in 63 studies (10.70%) that addressed osteoporosis in postmenopausal women. It is noteworthy that a relatively small number of registered clinical trials have explored antibodies for conditions such as endometriosis (3, 0.51%), genital herpes (1, 0.17%), ovarian insufficiency (1, 0.17%), and contraception (1, 0.17%). Figure 7 illustrates the distribution of clinical trials according to indications.

### 3.5 Targets

All the developing antibody-related drugs that are currently undergoing clinical trials in gynecology were cataloged systematically. The developed antibody-related drugs in gynecological clinical trials are summarized in Table 2. Currently, there are 44 antibodies in gynecological clinical development, but approximately 34% act on just 5 validated and novel targets. Predominantly, mesothelin emerged as the most prevalent (4, 9.09%), followed by programmed cell death protein 1 (PD-1) (3, 6.82%), folate receptor *α* (FRα) (3, 6.82%), receptor tyrosine-protein kinase erbB-2 (HER2) (3, 6.82%), and T cell immunoreceptor with immunoglobulin and tyrosine-based inhibitory motif domain (TIGIT) (2, 4.55%).

**Table 2:** Target and indication of developing antibody-related drugs in gynecological clinical trials.

Antibody type/name	Target	Phase	Trial number	Study Status	Indication
Canonical					
Balstilimab	PD-1	Phases 1–2	NCT03495882	Completed	Cervical cancer
		Phases 1–2	NCT03104699	Completed	Cervical cancer
		Phase 2	NCT03894215	Not recruiting	Cervical cancer
		Phase 1	NCT06095674	Not recruiting	Cervical cancer
Oregovomab	CA125	Phase 3	NCT04498117	Not recruiting	Ovarian cancer
		Phases 1–2	NCT04938583	Recruiting	Ovarian cancer
		Phase 2	NCT01616303	Completed	Ovarian cancer
		Phase 2	NCT05335993	Not recruiting	Ovarian cancer
BAT1308	PD-1	Phases 2–3	NCT06321068	Recruiting	Endometrial cancer
		Phases 2–3	NCT06123884	Not recruiting	Cervical cancer
ZB-06	CD52g	early phase1	NCT04731818	Completed	Contraception
Zalifrelimab	CTLA4	Phases 1–2	NCT03495882	Completed	Cervical cancer
Utomilumab	4-1BB	Phases 1–2	NCT03318900	Completed	Ovarian cancer
Tiragolumab	TIGIT	Phase 2	NCT04300647	Not recruiting	Cervical Cancer
Sirexatamab	DKK1	Phase 2	NCT03395080	Completed	Multiple gynecological cancers
Setrusumab	SOST	Phase 2	NCT01406548	Completed	Osteoporosis in postmenopausal women
Rulonilimab	PD-1	Phase 2	NCT06226350	Completed	Cervical cancer
Ociperlimab	TIGIT	Phase 2	NCT04693234	Completed	Cervical cancer
Murlentamab	AMHRII	Phase 1	NCT02978755	Completed	Ovarian cancer
MK-4830	ILT4	Phase 2	NCT05446870	Not recruiting	Ovarian cancer
Matuzumab	EGFR	Phase 2	NCT00073541	Completed	Ovarian cancer
Mapatumumab	TRAIL-R1	Phases 1–2	NCT01088347	Completed	Cervical cancer
Magrolimab	CD47	Phase 1	NCT03558139	Completed	Ovarian cancer
HMI-115	PRLR	Phase 2	NCT05101317	Recruiting	Endometriosis
HDIT101	Glycoprotein B	Phase 2	NCT04165122	Completed	Herpes genitalis
Ganitumab	IGF-1R	Phase 2	NCT00719212	Completed	Multiple gynecological cancers
EMD 273066	EPCAM	Phase 1	NCT00132522	Completed	Ovarian cancer
CDX-1140	CD40	Phase 2	NCT05231122	Recruiting	Ovarian cancer
Carotuximab	endoglin	Phase 2	NCT01381861	Completed	Multiple gynecological cancers
Amatuximab	Mesothelin	Phase 1	NCT00325494	Completed	Ovarian cancer
NP137	netrin-1	Phases 1–2	NCT04652076	Not recruiting	Multiple gynecological cancers
ADCs					
STRO-002	FRα	Phase 1	NCT03748186	Recruiting	Multiple gynecological cancers
		Phases 2–3	NCT05870748	Recruiting	Multiple gynecological cancers
		Phase 1	NCT05200364	Recruiting	Ovarian cancer
Anetumab Ravtansine	Mesothelin	Phase 2	NCT03587311	Not recruiting	Multiple gynecological cancers
		Phase 1	NCT02751918	Completed	Ovarian cancer
HS-20089	B7-H4	Phase 2	NCT06014190	Recruiting	Multiple gynecological cancers
IMGN151	FRα	Phase 1	NCT05527184	Recruiting	Multiple gynecological cancers
Trastuzumab duocarmazine	HER2	Phase 2	NCT04205630	Completed	Endometrial cancer
DP303c	HER2	Phase 2	NCT04828616	Not recruiting	Ovarian cancer
RC88	Mesothelin	Phase 2	NCT06173037	Not recruiting	Multiple gynecological cancers
Farletuzumab ecteribulin	FRα	Phase 2	NCT05613088	Recruiting	Multiple gynecological cancers
Raludotatug deruxtecan	CDH6	Phase 1	NCT04707248	Recruiting	Ovarian cancer
DMOT4039A	Mesothelin	Phase 1	NCT01469793	Completed	Ovarian cancer
DMUC4064A	MUC16	Phase 1	NCT02146313	Completed	Ovarian cancer
BNT323/DB-1303	HER2	Phase 3	NCT06340568	Not recruiting	Endometrial cancer
Fc-fusion protein					
Batiraxcept	GAS6	Phase 1	NCT05826015	Not recruiting	Endometrial cancer
		Phases 1–2	NCT04019288	Not recruiting	Multiple gynecological cancers
		Phase 1	NCT03639246	Completed	Ovarian cancer
SL-172154	SIRPα/CD47	Phase 1	NCT05483933	Not recruiting	Multiple gynecological cancers
		Phase 1	NCT04406623	Completed	Multiple gynecological cancers
SHR-1701	PD-L1/TGF-β	Phase 3	NCT05179239	Recruiting	Cervical cancer
Bispecific					
Navicixizumab	VEGF/DLL4	Phase 3	NCT05043402	Not recruiting	Multiple gynecological cancers
		Phase 1	NCT03030287	Completed	Multiple gynecological cancers
ZG005	PD-1/TIGIT	Phases 1–2	NCT06241235	Recruiting	Cervical cancer
Ubamatamab	CD3/MUC16	Phases 1–2	NCT03564340	Recruiting	Multiple gynecological cancers
REGN5668	MUC16xCD28	Phases 1–2	NCT04590326	Recruiting	Multiple gynecological cancers
Vudalimab	PD-1/CTLA-4	Phase 2	NCT05032040	Recruiting	Multiple gynecological cancers
Canonical					
Balstilimab	PD-1	Phases 1–2	NCT03495882	Completed	Cervical cancer
		Phases 1–2	NCT03104699	Completed	Cervical cancer
		Phase 2	NCT03894215	Not recruiting	Cervical cancer
		Phase 1	NCT06095674	Not recruiting	Cervical cancer
Oregovomab	CA125	Phase 3	NCT04498117	Not recruiting	Ovarian cancer
		Phases 1–2	NCT04938583	Recruiting	Ovarian cancer
		Phase 2	NCT01616303	Completed	Ovarian cancer
		Phase 2	NCT05335993	Not recruiting	Ovarian cancer
BAT1308	PD-1	Phases 2–3	NCT06321068	Recruiting	Endometrial cancer
		Phases 2–3	NCT06123884	Not recruiting	Cervical cancer
ZB-06	CD52g	early phase1	NCT04731818	Completed	Contraception
Zalifrelimab	CTLA4	Phases 1–2	NCT03495882	Completed	Cervical cancer
Utomilumab	4-1BB	Phases 1–2	NCT03318900	Completed	Ovarian cancer

## 4 Discussion

Since 1996, research into antibodies has significantly expanded in the field of gynecology. Innovative clinical trials, such as umbrella and basket trials, have been implemented to advance the development of antibody-related drugs in the field of gynecology. This study identified two basket trials (NCT04965519 and NCT03827837) and two umbrella trials (NCT03699449 and NCT03574779). Basket trials are characterized by the evaluation of targeted therapy for multiple diseases that share common molecular alterations. Umbrella trials involve the assessment of multiple targeted therapies within a single disease and can be further classified into various subgroups (17). The advantage of basket trials lies in the ability to test smaller sample sizes for a given tumor type, which facilitates research in less common tumor types. By contrast, umbrella trials allow for a more comprehensive analysis of treatment for a specific tumor type (18). The application of these novel clinical trials in gynecology is anticipated to accelerate the evaluation and approval of antibody-related drugs and promote molecular-based individualized treatment strategies (19, 20). In addition, innovative forms, such as ADCs, bispecific antibodies, and Fc-fusion proteins, have gained prominence and are the subjects of extensive investigation. In this review, studies were divided into four distinct groups according to the development of antibody-related drugs.

### 4.1 Canonical antibodies

Canonical antibodies exhibit a complete antibody structure, which contains two light chains and two full-length heavy chains (21). According to research, these antibodies represent more than 80% of all clinical trials in the field of gynecology.

Since the approval of ipilimumab in 2011, immune checkpoint inhibitors have significantly advanced the treatment of gynecological malignancies (22). In this field, significant inhibitors encompass PD-L1, PD-1, CTLA-4, and LAG-3 (23), among which PD-1/PD-L1 is the most extensively studied (24). Pembrolizumab and dostarlimab have been approved by the FDA to treat gynecological cancers (21). An increasing number of real-world studies contribute to the expanding body of evidence that supports the use of PD-1 inhibitors for recurrent or advanced gynecologic cancers. In a multi-center retrospective cohort study, Korean patients with recurrent endometrial cancer were administered pembrolizumab in combination with lenvatinib for a median duration of 4.5 cycles. During this period, the disease control rate was 76.2% (95% CI, 61.9–88.1), and the best objective response rate was 23.8% (95% CI, 11.9–38.1) (25). The most common treatment-related adverse events included fatigue, hypertension, and hypothyroidism. Additionally, another retrospective study suggested that a lower dosage of pembrolizumab represents a cost-effective and efficacious treatment strategy for patients with refractory gynecologic cancer (26).

The vascular endothelial growth factor (VEGF) serves as a crucial mediator in the process of neovascularization, which is predominantly secreted by cancer cells (27). Bevacizumab is the first anti-angiogenic agent approved for oncological use. Numerous clinical trials have demonstrated the efficacy of bevacizumab in the treatment of gynecologic malignancies (28). There is an increasing body of evidence that supports its efficacy and safety in routine clinical practice. In a retrospective study, bevacizumab was found to be effective and well-tolerated in the real-world treatment of ovarian cancer (29). Furthermore, a multi-institutional retrospective cohort study indicated that bevacizumab maintenance therapy prolonged progression-free survival for metastatic, recurrent, or persistent cervical cancer (30).

In addition, canonical antibodies are also used in the treatment of postmenopausal osteoporosis (31). Notably, denosumab, a fully human antibody targeting RANK-L (32), and romosozumab, a humanized monoclonal antibody targeting sclerostin (SOST) (33), are utilized in this therapeutic context.

### 4.2 ADCs

ADCs have emerged as one of the most promising therapeutic modalities in oncology due to their ability to provide tumor cells with chemotherapy in a more targeted and safer manner (34, 35). ADCs comprise a highly selective antibody, a cytotoxic payload, and a linker connecting the two components (36). Currently, several ADCs have acquired FDA approval for the treatment of gynecologic cancers, including mirvetuximab soravtansine for ovarian cancer and tisotumab vedotin for cervical cancer (37). Although no ADCs have been approved for endometrial cancer, 8 clinical trials (NCT04205630, NCT06132958, NCT03836157, NCT04251416, NCT06340568, NCT03835819, NCT03832361, and NCT04585958) have been identified to address this unmet critical need. Notably, mirvetuximab soravtansine is currently being evaluated in two phase II clinical trials for endometrial cancer (NCT03835819 and NCT03832361).

Fam-trastuzumab deruxtecan-nxki (T-DXd) contains a topoisomerase I inhibitor payload, a cleavable tetrapeptide linker, and the anti-HER2 antibody trastuzumab (38). On 5 April 2024, the approval of T-DXd was accelerated by the FDA to treat adults with unresectable or metastatic HER2-positive (IHC3+) solid tumors. Previous clinical trials have suggested its potential efficacy in patients with endometrial cancer (39). In the STATICE trial, patients with endometrial cancer expressing HER2 were categorized into HER2-low and HER2-high expression groups. The overall response rate was 70.0% (95% CI, 34.8–93.3) for the HER2-low group and 54.5% (95% CI, 32.2–75.6) for the HER2-high group. These results indicated that T-DXd is effective in patients with endometrial cancer, irrespective of the HER2 expression status (40). Furthermore, the combination of T-DXd and olaparib was assessed in HER2-expressing cancers, including an extension to patients with endometrial cancer (NCT04585958).

Sacituzumab govitecan, ADCs against trophoblast cell surface antigen-2 (Trop-2), has demonstrated its potential efficacy in the treatment of endometrial cancer (41). In a Phase I/II basket study, the objective response rate was 22.2% (95% CI, 6.4–47.6) in the endometrial cancer cohort (42). Currently, a Phase II study of sacituzumab govitecan in patients with persistent or recurrent endometrial carcinoma is ongoing (NCT04251416).

However, in this context, clinical data on other ADCs remain limited.

### 4.3 Bispecific antibodies

Bispecific antibodies are designed to simultaneously bind to separate antigenic epitopes or distinct antigens (43). Compared with traditional antibodies, bispecific antibodies exhibit greater specificity, which contributes to improved therapeutic efficacy and safety, while reducing the likelihood of adverse reactions (44). This characteristic makes bispecific antibodies a promising strategy in cancer therapy (45). An increasing number of bispecific antibodies with diverse mechanisms are currently under development for oncological applications, including checkpoint inhibitors (CPIs), T-cell engagers (TCEs), natural killer cell engagers (NKCEs), and immune cell engagers (ICEs) (46). In the realm of gynecological cancer, the majority of bispecific antibodies in registered clinical trials are concentrated on checkpoint inhibition. Notably, catumaxomab (NCT00189345, NCT00563836), navicixizumab (NCT05043402, NCT03030287), REGN5668 (NCT04590326), and ubamatamab (NCT03564340) are identified as agents targeting CD3xEpCAM, VEGFxDLL4, MUC16xCD28, and CD3xMUC16, respectively. A selection of representative findings is described in this subsection.

Cadonilimab is a bispecific antibody against PD-1 and CTLA4, which has completed two Phase II studies in patients with metastatic or recurrent cervical cancer (NCT04380805, NCT04868708). The findings of these studies indicated that cadonilimab may provide patients with substantial therapeutic benefits (47, 48). In China, cadonilimab was approved in June 2022 for the treatment of metastatic cervical cancer based on these promising outcomes (49). Furthermore, several clinical studies involving cadonilimab are currently ongoing (NCT05687851, NCT05824494, NCT06066216, NCT04982237, NCT05227651, and NCT05932212).

Navicixizumab is a bispecific agent that targets both DLL4 and VEGF (50). A Phase 1a study of navicixizumab provided preliminary evidence of anti-tumor activity in ovarian cancer, with manageable toxicity (51). A subsequent Phase 1b study combining navicixizumab with paclitaxel (NCT03030287) demonstrated an ORR of 43.2% (95% CI, 28.3–59.0) in patients with platinum-resistant ovarian cancer. Furthermore, 90.9% of patients experienced treatment-related adverse events, including hypertension, fatigue, and headaches (52). A Phase III trial to assess the efficacy and safety of navicixizumab in the treatment of ovarian cancer is currently underway (NCT05043402).

### 4.4 Fc fusion proteins

Fc fusion proteins contain the Fc domain of an immunoglobulin (IgG) and a desired linked protein (53). Compared to traditional antibodies, Fc fusion proteins exhibit an extended half-life, multiple targeting capabilities, and superior specificity (54). Currently, there are 13 studies on Fc fusion proteins in the field of gynecology. This report highlights the bifunctional fusion proteins Bintrafusp alfa (NCT04246489, NCT04551950), SHR-1701 (NCT05179239), and SL-172154 (NCT05483933, NCT04406623).

Bintrafusp alfa is a bifunctional fusion protein that comprises an extracellular domain of TGF-*β*receptor II fused to a human IgG1 monoclonal antibody against PD-L1 (55). This agent has completed both Phase 1 and Phase 2 clinical trials in patients with metastatic or recurrent cervical cancer (NCT04551950, NCT04246489). The findings of these studies have confirmed the safety profile and clinical efficacy of Bintrafusp alfa. Anemia is the most common treatment-related adverse event (56, 57).

SHR-1701 is a bifunctional fusion protein that comprises an extracellular domain of TGF-β II and an antibody targeting PD-L1 (58). Recent data have indicated that SHR-1701 has an ORR of 15.6% in patients with metastatic or recurrent cervical cancer. Additionally, 11 patients (34.4%) experienced treatment-related adverse events of grade 3 or grade 4 severity. Consequently, SHR-1701 may be a potential therapeutic option for patients with cervical cancer (59). Currently, the safety and efficacy of SHR-1701 are being evaluated in a Phase 3 clinical trial (NCT05179239).

SL-172154 is a hexameric fusion protein that comprises SIRPα and CD40L domains connected by an inert Fc linker. This agent has completed a Phase 1 clinical trial in ovarian cancer patients (NCT04406623). Additionally, a subsequent Phase 1 trial is currently underway, which targets patients with platinum-resistant ovarian cancer (NCT05483933).

### 4.5 Challenges

#### 4.5.1 Subcutaneous delivery of antibodies

Historically, antibody-related drugs are delivered by IV injection due to their inherent physicochemical and biological characteristics (60). However, this method has several disadvantages, including pain, low patient tolerance, and elevated costs. Consequently, there has been a progressive transition in the administration route of antibody-based therapeutics from IV to SC injection. This shift is attributed not only to the reduced administration time but also to the feasibility of home-based administration (61). In recent years, approximately 30% of the approved antibody-related drugs have been administered through SC injection (62). Although several drugs have reached clinical trials, only denosumab and romosozumab have been approved by the EMA and FDA to treat postmenopausal osteoporosis (63).

There were no statistically significant differences in safety and immune response between intramuscular and SC administration routes, according to a Phase 1 clinical trial (NCT00058435) (64). Similarly, the findings of another Phase 1 clinical study (NCT00103545) were also promising (65). However, a subsequent Phase 3 trial (NCT00418574) indicated that patients with ovarian cancer in first remission received no therapeutic benefits from abagovomab (66).

Dalantercept, a recombinant fusion protein designed to target the activin receptor-like kinase 1 (ALK1) receptor, was evaluated in a Phase II clinical trial that comprised 28 patients with endometrial cancer. Patients were administered a dosage of 1.2 mg/kg subcutaneously every 3 weeks (NCT01642082). The majority of participants withdrew from the study primarily due to disease progression. It was concluded that dalantercept had inadequate efficacy as a single-agent treatment (67). Additionally, a separate Phase II trial that evaluated dalantercept in patients with ovarian and fallopian tube cancers failed to exhibit any objective response (68).

Hyaluronidase-zzxf/pertuzumab/trastuzumab is an SC injection that contains a fixed-dose combination of trastuzumab and pertuzumab, which has been approved for treating patients with HER2-positive breast cancer (69). Currently, a Phase 2/3 clinical trial is underway to assess the efficacy of hyaluronidase-zzxf/pertuzumab/trastuzumab in patients with HER2-positive endometrial serous carcinoma (NCT05256225).

Furthermore, a Phase 2 clinical trial is currently underway to assess the efficacy of HMI-115 in patients with endometriosis-related pain. Herein, HMI-115 is administered SC on a bi-weekly basis.

This study shows that the availability of antibody-related drugs for SC administration is limited in the field of gynecology. This is because this route of administration is hindered by challenges such as immunogenicity, high viscosity, and protein aggregation (70, 71). To enhance the SC delivery of antibodies, various strategies have been developed, including the combination of proprietary excipients and proteins to address the issues related to the ionic strength and hydrophobic regions of antibodies (60).

Overall, the demand for SC antibodies is high on the market. However, significant obstacles persist and the SC administration is making ongoing advancements.

#### 4.5.2 Potential targets

According to the results, the hottest and most potential targets for developing antibody-related drugs in gynecology are mesothelin, PD-1, FRα, HER2, and TIGIT. Mesothelin is a membrane-bound surface glycoprotein that is highly expressed in ovarian cancer, pancreatic adenocarcinoma, mesothelioma, and several other malignancies. However, its expression is limited in normal tissues (72). Furthermore, mesothelin plays an important role in cell adhesion, drug resistance, and tumor metastasis, which makes it a potential therapeutic target for ovarian cancer (73). Examples are amatuximab (NCT00325494), anetumab ravtansine (NCT03587311, NCT02751918), and RC88 (NCT06173037).

PD-1 is a checkpoint protein that belongs to the CD28 family. It is predominantly expressed in activated CD4 + T cells, CD8 + T cells, and peripheral B cells (74). The PD-1 signaling pathway plays a significant role in the tumor microenvironment of various malignancies and contributes to T cell inactivation and depletion, which facilitates the evasion of antitumor immunity (75). Consequently, PD-1 is a promising target for therapeutic strategies. Examples are balstilimab (NCT03495882, NCT03104699, NCT03894215, NCT06095674), BAT1308 (NCT06321068, NCT06123884), and rulonilimab (NCT06226350).

FRα is a glycoprotein attached to the membrane and is encoded by the FOLR1 gene (76). It is critically involved in cell proliferation, DNA synthesis, and intracellular signaling, which are fundamental to tumorigenesis. The receptor is a promising target for the development of anticancer drugs due to its overexpression in various solid tumors, such as ovarian, lung, and breast cancers (77). Examples are STRO-002 (NCT03748186, NCT05870748, and NCT05200364), IMGN151 (NCT05527184), and farletuzumab ecteribulin (NCT05613088).

HER2 is a constituent of the EGFR family of receptor tyrosine kinases (78). Aberrations of the HER2 gene, including mutations, deletions, and amplifications, have been identified in ovarian, cervical, and endometrial cancers (79). Examples are trastuzumab duocarmazine (NCT04205630), DP303c (NCT04828616), and BNT323/DB-1303 (NCT06340568).

TIGIT is a newly recognized immune checkpoint. It is predominantly expressed in natural killer cells, regulatory T cells, CD4 + T cells, CD8 + T cells, and tumor-infiltrating lymphocytes (80). TIGIT is associated with the exhaustion of NK cells *in vivo* and is observed in individuals with various types of cancer (81). Examples are tiragolumab (NCT04300647) and ociperlimab (NCT04693234).

## 5 Conclusion

This study provides a thorough review of clinical trials involving antibody-related drugs in the field of gynecology. The analysis revealed a growing prevalence of ADCs, bispecific antibodies, and Fc-fusion proteins. It has been found that these therapeutic modalities play a crucial role in advancing gynecological oncology. Meanwhile, it also explores the challenges associated with SC administration. The findings of this review may provide valuable insights and new ideas for future research on antibodies in gynecology.

## Data Availability

The original contributions presented in the study are included in the article/supplementary material, further inquiries can be directed to the corresponding author.
